# Thermosensory Perceptual Learning Is Associated with Structural Brain Changes in Parietal–Opercular (SII) Cortex

**DOI:** 10.1523/JNEUROSCI.1316-17.2017

**Published:** 2017-09-27

**Authors:** Hiroaki Mano, Wako Yoshida, Kazuhisa Shibata, Suyi Zhang, Martin Koltzenburg, Mitsuo Kawato, Ben Seymour

**Affiliations:** ^1^Center for Information and Neural Networks, National Institute of Information and Communications Technology, Osaka, Japan 565-0871,; ^2^Advanced Telecommunications Research Institute International, Seika-cho, Soraku-gun, Kyoto 619-0288, Japan,; ^3^Graduate School of Environmental Studies, Nagoya University, Furo-cho, Chikusa-ku, Aichi 464-0814, Japan,; ^4^Institute of Neurology, University College London, London WC1N 3BG, United Kingdom, and; ^5^Computational and Biological Learning Laboratory, Department of Engineering, University of Cambridge, Cambridge CB2 1PZ, United Kingdom

**Keywords:** interoception, pain, perceptual learning, thermal, thermosensory, VBM

## Abstract

The location of a sensory cortex for temperature perception remains a topic of substantial debate. Both the parietal–opercular (SII) and posterior insula have been consistently implicated in thermosensory processing, but neither region has yet been identified as the locus of fine temperature discrimination. Using a perceptual learning paradigm in male and female humans, we show improvement in discrimination accuracy for subdegree changes in both warmth and cool detection over 5 d of repetitive training. We found that increases in discriminative accuracy were specific to the temperature (cold or warm) being trained. Using structural imaging to look for plastic changes associated with perceptual learning, we identified symmetrical increases in gray matter volume in the SII cortex. Furthermore, we observed distinct, adjacent regions for cold and warm discrimination, with cold discrimination having a more anterior locus than warm. The results suggest that thermosensory discrimination is supported by functionally and anatomically distinct temperature-specific modules in the SII cortex.

**SIGNIFICANCE STATEMENT** We provide behavioral and neuroanatomical evidence that perceptual learning is possible within the temperature system. We show that structural plasticity localizes to parietal–opercular (SII), and not posterior insula, providing the best evidence to date resolving a longstanding debate about the location of putative “temperature cortex.” Furthermore, we show that cold and warm pathways are behaviorally and anatomically dissociable, suggesting that the temperature system has distinct temperature-dependent processing modules.

## Introduction

Despite significant progress in our understanding of the peripheral mechanisms of temperature sensation (Caterina et al., 1997; Bautista et al., 2007; Mishra et al., 2011; Pogorzala et al., 2013; Vriens et al., 2014; Ran et al., 2016), central mechanisms remain much less clear. That humans can detect temperature changes of a fraction of a degree (Kenshalo et al., 1960; Dyck et al., 1971; Johnson et al., 1973; Chen et al., 1996), bearing in mind the relatively broad response profile of thermoreceptors, strongly points to the existence of a specific “temperature cortex,” but its anatomical location remains unresolved.

One view is that parietal–opercular cortex (SII) supports temperature perception via ventrolateral thalamic relay of thermally responsive spinal afferents (Vriens et al., 2014). This view accords with temperature as an exteroceptive sense (an inference about the outside world) similar to other somatosensory modalities such as touch and vibration. An alternative view proposes that the posterior insula (PI) incorporates temperature cortex, via medial thalamic nuclei (including VMPo), as part of a broader interoceptive cortex that also accommodates pain, itch, and pleasant touch (Craig, 2002; Hua et al., 2005). This view draws on a view as temperature perception as an inference about the physiological state of the body, along with other sensory modalities that have intrinsic motivational value through a direct link with homeostasis (e.g., behavioral thermoregulation).

Cortical stimulation of both parietal–opercular and PI can induce thermal sensations, with warmth being the more common sensation (Ostrowsky et al., 2002; Isnard et al., 2004, 2011; Mazzola et al., 2006; Mazzola et al., 2012). Human PI lesions have been reported as causing thermal anesthesia and impairing thermal detection in humans (Birklein et al., 2005; Cattaneo et al., 2007; Baier et al., 2014), but in rodents, SI lesions have been shown to impair cold discrimination (Milenkovic et al., 2014) and human SI disruption with tDCS impairs bilateral cold detection (Oliviero et al., 2005; Grundmann et al., 2011). Awake electrocortical responses have suggested that SII better codes warmth and PI pain (Frot et al., 2007), but both regions have been observed to respond to warmth in fMRI studies (Davis et al., 1998; Bornhövd et al., 2002; Moulton et al., 2012). Good neuroimaging evidence exists for topographic cold responses in PI (Craig et al., 2000; Hua et al., 2005) and cold responses have also been localized to PI in MEG data (Maihöfner et al., 2002), although recent combined EEG–MEG data have suggested a source in SII (Fardo et al., 2017).

Together, these studies have led to a consensus favoring PI as thermosensory cortex proper (Craig, 2002, 2011). Recently, however, high-density human intracortical electrophysiology suggested that PI may instead support a multimodal sensory integration zone, rather than holding modality-specific representations (Liberati et al., 2016). Therefore, whereas it may have a prominent role in homeostatic functions relating to temperature, whether PI acts as a primary locus for discriminative thermal perception is unresolved.

A key lacunae in the evidence to date is any neuroanatomical mapping of fine temperature discrimination. As the prototypical feature of cortical sensory processing, it almost certainly depends on cortical information processing across a population of thermoreceptors with different tuning functions (Pogorzala et al., 2013). In a manner similar to other discriminative sensory modalities such as vision and hearing, fine discriminative processing of sensory afferent signals can be considered the primary function of a putative thermosensory cortex. One method to identify a cortical locus of discrimination is to look for structural changes associated with perceptual learning (Zatorre et al., 2012). Although thermosensory perceptual learning has not been described previously, in the visual domain, it has been shown that as little as 5 d of repetitive training can lead to behavioral improvements and associated gray matter (GM) increases in the corresponding cortical sensory area (Ditye et al., 2013). Following this approach, we trained subjects to discriminate very small changes in either warm or cold temperatures and probed corresponding anatomical brain changes with structural neuroimaging.

## Materials and Methods

### 

#### Participants

Twenty-four healthy subjects completed the study (8 females, age: 24.5 ± 6.03 years). This does not include 10 subjects who started the experiment but could not complete training due to technical failure of the thermal stimulator during perceptual training (requiring a replacement stimulator to be shipped from abroad) and were therefore excluded. All subjects had normal or corrected-to-normal vision and were screened for a history of psychiatric or neurological conditions. All subjects gave a written informed consent that was approved by the ethics committee of Advanced Telecommunication Research Institute International (ATR, Kyoto, Japan) and the National Institute of Information and Communications Technology (NICT, Tokyo, Japan).

#### Thermal stimuli

We used a contact thermal stimulator (ATS PATHWAY; Medoc) to deliver thermal stimuli. The thermode was attached to the lateral aspect of the left or right upper calf using a Velcro strap, the stimulation sites were marked on the first day, and the same site was used for all subsequent experimental sessions. Between experimental sessions, the thermode was kept at a resting temperature of 30°C and changed to the baseline temperature (25°C or 39°C) just before each experimental session.

#### Experimental procedure

Each of the 24 subjects attended the experiment on 9 separate days: pretraining MRI scanning (day 1), pretraining behavioral test session (day 2), 5 d of training sessions (days 3–7), posttraining behavioral test session (day 8), and a posttraining MRI scanning (day 9). Training and test sessions were completed within a maximum of 14 d to minimize forgetting effects in perceptual learning. Some subjects performed pre-/posttraining behavioral tests and scanning on the morning and afternoon of the same day for logistical reasons.

##### Thermal discrimination task.

**Figure 1.**
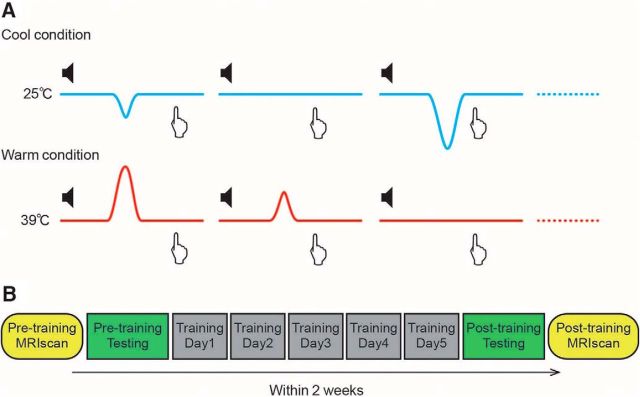
Thermal detection task. ***A***, Subjects performed a simple detection task in which they had to press a button if they felt a small decrease (from a 25°C baseline in the cold condition) or increase (from a 39°C baseline in the warm condition) occurring with 50% probability. The phasic temperature changes were of four different magnitudes to create a range of difficulties and were calibrated to each subject beforehand (see Materials and Methods). The start of each trial was signaled by a message on the computer monitor and the timing of the possible temperature change was cued by an auditory tone 1.5 s in advance. ***B***, Experimental schedule: subjects underwent 5 d of training with a specific temperature and laterality. Before and after training, they performed behavioral testing on all temperatures/lateralities and underwent structural and functional imaging.

We performed a one-interval thermal-pulse detection task in which subjects were required to report the presence of a small reduction (from the 25°C cool baseline) or increase (from the 39°C warm baseline) in temperature for cold and warm detection, respectively (see Fig. 1). These thermal pulses occurred in 50% of trials and across four different magnitudes, thus making four different levels of difficulty.

At the beginning of each trial, subjects heard a small tone through their headphones, accompanied by a visual message, “Press the button if you feel a pulse,” displayed on a computer monitor for 500 ms. Then, the thermode either delivered the pulse stimulus or continued at baseline. If they felt a thermal pulse, then they responded by pressing a button within 3.5 s. If they felt no pulse, then they were instructed not to press the button. There was no feedback (i.e., whether the detection was correct) given to the subjects. Each session had 200 trials consisting of 100 trials with thermal pulse delivery (25 for each level of difficulty) and 100 trials with no pulse. The order of pulse and no-pulse trials was pseudorandomized. Each session took ∼15 min.

##### Calibration across subjects.

The four levels of difficulty were set individually for each subject before the experiment was performed because there is significant between-subject variability in discriminative performance, so we aimed to equate performance approximately across subjects. In this calibration procedure, subjects received a range of thermal pulses from 0.2°C to 1.5°C (0.2°C, 0.3°C, 0.5°C, 0.7°C, 0.9°C, 1.1°C, 1.3°C, and 1.5°C). We chose the 4 adjacent temperatures that gave an accuracy (i.e., sensitivity index, *d*′, see below) closest to 1.5 (typically this corresponds to ∼75% correct, with 50% being chance). The most common set of temperatures pulses was 0.5°C, 0.7°C, 0.9°C, and 1.1°C.

##### Pretraining testing.

After the calibration procedure, subjects performed the pretraining behavioral testing of both warming and cooling on both right and left leg. Specifically, they performed two sessions of cold testing on the left leg, two sessions of cold on the right leg, two sessions of warm training on the left leg, and two on the right leg. The order of performing each was balanced and randomized across subjects, but identical in the posttraining session.

##### Training.

For the training sessions, subjects were assigned randomly to be trained on one of four task conditions. Randomization was determined before the start of the entire experiment, but blinded to experimenters until after pretraining test to avoid bias (the pretest discriminative accuracy of the trained temperature/laterality was not different from the nontraining temperature/lateralities). On each training day, subjects performed 4 sessions of their allocated temperature/laterality over 5 d (i.e., 800 trials per day in total, lasting approximately 1 h).

##### *Post*training testing.

After training, the subjects performed posttraining task on both temperatures and lateralities exactly as in the pretraining test.

#### MRI acquisition

Structural brain images were obtained in an MRI scanner before and after the experimental task sessions. Resting-state fMRI scans were also collected, during which subjects were instructed to keep looking at a central fixation point, to keep still, and to stay awake. We also performed an fMRI task with small fixed pulses in warm and cool temperatures. Postexperimental analysis revealed the presence of radiofrequency noise introduced by the operation of the thermal stimulator, creating artifact that corrupted the images in a way that was correlated with the task, so these data were discarded. We also collected diffusion-weighted images. This was intended to generate pilot data for a future study of white matter (WM) changes associated with learning.

All scanning was performed on a 3.0 T MRI Scanner (3T Magnetom Trio with TIM system; Siemens) equipped with echo-planar imaging (EPI) capability and a standard 12-channel phased array head coil. Subjects remained supine and wore MR-compatible headphones.

A 6 min resting-state functional MRI (rsfMRI) scan consisting of 145 volumes was acquired using a single-shot EPI gradient echo T2*-weighted pulse sequence with the following parameters: TR = 2500 ms, TE = 30 ms, FA = 80 degrees, BW = 2367 Hz, FOV = 192 × 192 mm (covering the whole brain), acquisition matrix = 64 × 64, 37–41 axial slices with an ascending slice order of 2.5 mm slice thickness with 0.5 mm interslice gap. In parallel with the rsfMRI scan, cardiac pulsation and respiratory waveform were monitored with a photoplethysmography probe attached to the distal end of a finger on the left hand and with a respiration belt strapped around the upper abdomen and recorded with a sampling rate of 50 Hz.

A high-resolution three-dimensional volumetric acquisition of T1-weighted structural MRI scan was collected using a MPRAGE pulse sequence: TR = 1.07 ms, TE = 3.06 ms, time of inversion = 900 ms, FA = 9 degrees, BW = 230 Hz, FOV = 256 × 256 mm, 208 sagittal slices of 1 mm slice thickness with no interslice gap, acquisition matrix = 256 × 256.

#### Data analysis

##### Behavioral analysis.

Accuracy was measured by calculating the *d*′ in the standard manner: *d*′ = *Z*(hit rate) − *Z*(false alarm rate). The *d*′ was then used a summary statistic in ANOVA and *t* tests as appropriate.

##### Voxel-based morphometry (VBM) analysis.

VBM analysis was performed with statistical parametric mapping SPM8 (Wellcome Trust Centre for Neuroimaging, UCL, London, UK; http://www.fil.ion.ucl.ac.uk/spm/), its default plug-in toolbox, diffeomorphic anatomical registration using exponentiated Lie algebra (DARTEL) (Ashburner, 2007), and their extension, VBM8 (Christian Gaser, Department of Psychiatry, University of Jena, Germany; http://dbm.neuro.uni-jena.de/vbm/) on MATLAB (The MathWorks). T1-weighted images were fed into this analysis pathway and we applied a specialized framework for longitudinal analysis in VBM8 consisting of the following procedures.

To study changes across time within the same subject, the obtained subject-specific images from pretraining and posttraining MRI scanning were registered in the individual subject space and the mean image was generated. The original images were realigned into the mean image to avoid the occurrence of potential bias due to asymmetry in pairwise image registration. A correction for intensity inhomogeneity was performed for the realigned images. The derived images were segmented into GM and WM based on an adaptive Maximum A Posterior (MAP) technique. The GM and WM images were spatially normalized and registered to IXI550 MNI152 space (IXI-database; http://brain-development.org/ixi-dataset/) with a manner of high-dimensional deformation. These images were smoothed with an 8 × 8 × 8 mm FWHM Gaussian kernel and used for the further statistical analyses.

Interaction between the differences in trained task condition (cool and warm pulse detection) and training effect (pretraining and posttraining) were tested for statistical significance in a flexible factorial ANOVA with a threshold at uncorrected *p* < 0.001 after application of a small-volume correction encompassing bilateral SII (OP1, OP2, OP3, and OP4) and PI (Ig1, Ig2, and Id1) regions as defined in the SPM Anatomy toolbox (Eickhoff et al., 2005).

We also did a *post hoc* analysis of the effect of laterality using cold and warm masks (at *p* < 0.005 uncorrected) to contrast directly contralateral minus ipsilateral effect sizes. This allowed us to group the effects of left- and right-trained subjects for each temperature. Finally, we also considered whether there might be warm- or cold-specific responses in SI cortex, so we performed a supplementary analysis using a mask from a probabilistic atlas (Geyer et al., 1999).

##### rsfMRI seed-based correlation analysis.

rsfMRI data were analyzed with SPM8 and the FMRIB Software Library (FSL; http://fsl.fmrib.ox.ac.uk/fsl/). The first five images were discarded to allow for T1 equilibration and the remaining images were corrected for physiological noise and cardiac and respiratory artifacts by applying the RETROICOR method (Glover et al., 2000). Slice timing was adjusted to the intermediate slice and all of the images were realigned to the first image of each scan with the estimated six rigid-body head motion parameters with SPM8. In addition, a wavelet-based despiking method (Patel et al., 2014) was applied to all the realigned images to attenuate a range of spurious variance related to abrupt head motions. Nonbrain structures such as skull and scalp surfaces were removed (Jenkinson et al., 2002; Smith, 2002) before the performance of boundary-based registration (Greve and Fischl, 2009) between the first image of the functional images and the corresponding T1-weighted structural image, followed by spatial normalization to the Linear ICBM Average Brain (ICBM152) Stereotaxic Registration Model (Mazziotta et al., 1995, 2001a, 2001b) with 12 degrees-of-freedom linear affine transformation. Smoothing was applied with an 8 × 8 × 8 mm FWHM Gaussian kernel and a temporal band-pass filter ranging from 0.01–0.08 Hz was applied.

Next, seed-based correlation analysis was applied. The seed ROIs for cold and warm conditions were defined by the VBM results on T1-weighted images (see “VBM results” section). Based on the average time course within each of the ROIs, connectivity was calculated as Pearson's correlation coefficient for all other voxels in the brain and then Fisher's *Z*-transformation was applied. Statistical analysis was performed to compare pretraining and posttraining effect for the cold seed ROI in the cold-trained subjects together with that for the warm seed ROI in the warm-trained subjects.

## Results

### Behavioral results

Twenty-four subjects performed a thermosensory perceptual learning experiment to identify improvements in accuracy in fine temperature discrimination in a one-interval detection task (without feedback) within warm (from 39°C) and cold (from 25°C) temperature domains. Thermal stimuli were delivered by a contact Peltier thermode applied to the left or right leg and subjects were required to identify the presence of a transient change in baseline temperature (cooling in the cold domain and warming in the warm domain) that occurred with 50% probability across four levels of difficulty determined by the magnitude of the phasic temperature change. At the start of the experiment, subjects were tested on discriminative accuracy for both warm and cold conditions on both right and left legs. Then, subjects were randomized into two groups: 12 subjects were trained to discriminate brief increases from a warm baseline temperature (39°C) and 12 subjects were training with to detect transient decreases from a cool baseline (25°C) (Fig. 1). Within these groups, subjects were randomized to be trained on either the left or right leg. Subjects performed the task for approximately 1 h on 5 d (over the course of ∼1 week) on their respective temperature and laterality. After training, they were retested on both temperatures and lateralities so that we could identify improvements in discriminative accuracy (*d*′) as a specific function of training. MRI scanning was done before and after the behavioral sessions to look for evidence of neural plasticity (see below).

**Figure 2.**
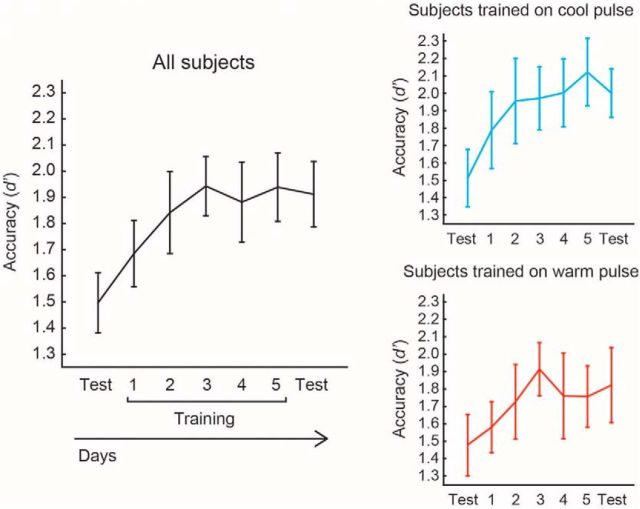
Performance over test and training sessions. Accuracy improved with training over time, when evaluated across all subjects (left) or restricted to within the cold-trained and warm-trained groups (right). Error bars indicate SEM.

Accuracy was improved as a function of training, with a significant increase in the *d*′ (Δ*d*′) of 0.44 across all subjects when comparing pretraining and posttraining performance on the temperature and laterality on which they were trained (one-sample *t* test, *n* = 24, *p* = 0.0005) (Fig. 2). The effect was more clear in the cold-training group (*n* = 12, Δ*d*′ = 0.49, *p* = 0.005) than in warm subjects (*n* = 12, Δ*d*′ = 0.40, *p* = 0.042).

**Figure 3.**
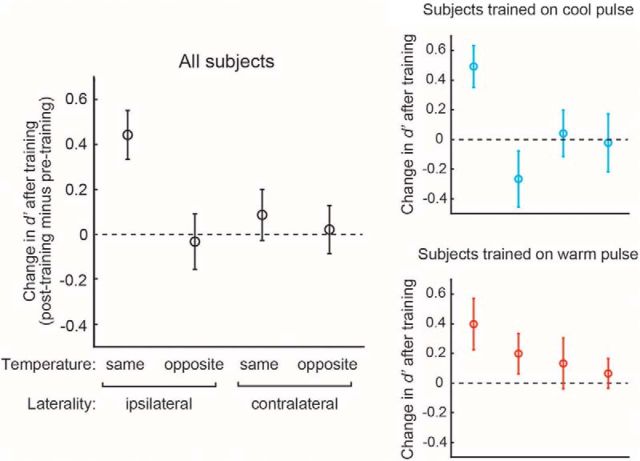
Perceptual detection accuracy. Left, Change in accuracy (*d′*) at the posttraining test session compared with pretraining across all subjects (*n* = 24). The *x*-axis refers to the temperature and laterality being tested, with “same, ipsilateral” referring to the trained temperature and laterality. Right, Same analysis, but split into the cold-trained (*n* = 12) and warm-trained (*n* = 12) subjects.

To probe the specificity of this effect, we compared the improvement in accuracy for the temperature/laterality on which they were trained with those on which they were not. Across all subjects, a two-way ANOVA (based on using the posttraining minus pretraining contrast as the summary statistic) revealed a main effect of temperature (*F* = 5.66, *p* = 0.019), but no significant main effect of laterality (*F* = 1.77, *p* = 0.1863) and a nonsignificant interaction (*F* = 3.26, *p* = 0.074) (Fig. 3). That is, the improvement in discriminative accuracy was restricted to the temperature, cold or warm, being trained.

To study this effect in more detail, we then looked separately at the cold- and warm-trained subjects. Cold subjects showed a main effect of temperature (*F* = 5.71, *p* = 0.021), no effect of laterality (*F* = 0.36, *p* = 0.549), and a marginally significant temperature × laterality interaction (*F* = 4.08, *p* = 0.0494) (Fig. 3, upper right). Warm subjects showed no main effect of temperature (*F* = 0.21, *p* = 0.375), no effect of laterality (*F* = 0.478, *p* = 0.187), and no significant temperature × laterality interaction (*F* = 0.052, *p* = 0.661) (Fig. 3, lower right). This suggests that the training effect is more robust for cold than warm temperatures.

**Figure 4.**
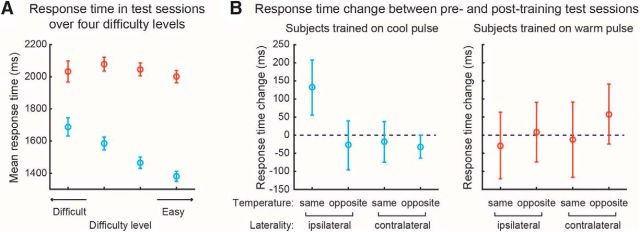
RTs. ***A***, RTs as a function of the difficulty of successfully detected stimuli across warm and cold trials. ***B***, Mean improvement from pretraining to posttraining (ΔRT) in cold-trained subjects (left) was 131.7 (*p* = 0.1619). Between-condition ANOVA identified a nonsignificant main effect of temperature (*p* = 0.158) and no temperature × laterality interaction (*p* = 0.223). In warm-trained subjects (right), there were no observable changes in RTs (ΔRT = −28.5 ms, *p* = 0.760) and no main effects or interactions.

Response times (RTs) were significantly faster for cold detection (mean = 1459 ms) than warm detection (mean = 2026 ms) (*t* test, *p* < 1*e* − 14), which is consistent with the notion that cold detection relies on myelinated A-delta fibers, whereas warm detection relies on unmyelinated C fibers. Figure 4*A* shows the RTs as a function of difficulty, illustrating that only cold detection shows longer RTs for correctly identifying the smaller, more difficult stimuli than the easier, larger stimuli. With respect to training, there was no difference in overall RTs between the pretraining and posttraining tests (ΔRT) when looking at all subjects and conditions (ΔRT = 8.1 ms, *p* = 0.8668) or just warm-trained subjects (ΔRT = 21.96 ms, *p* = 0.6820) or cold-trained subjects (ΔRT = 38.15 ms, *p* = 0.4752). Figure 4*B* looks specifically at RTs as a function of training and, although the overall pattern suggests a reduction in RT mirroring improvements in accuracy, these effects do not reach significance (see figure legend for statistics).

In conclusion, there was evidence for perceptual learning across both warm- and cold-trained subjects. Overall, this was specific to the temperature being trained and this effect was primarily driven by more robust learning in the cold-trained subjects, with learning present but less robust in the warm-trained subjects.

### Neuroimaging results

We next sought to identify brain regions associated with perceptual learning by comparing GM volume from structural T1-weighted MRI scans before and after training using VBM (Ashburner and Friston, 2000). An initial contrast of posttraining minus pretraining scans across all subjects did not identify any differences within an atlas-based mask that comprised bilateral SII and PI cortex as our regions of interest (see methods), or at whole brain level (with appropriate corrections for multiple comparisons). Based on the behavioral observation that learning was temperature specific, we therefore directly contrasted post- minus pretraining VBM maps between cold-trained and warm-trained subjects (i.e., to identify an interaction between the effect of training and temperature), regardless of laterality.

**Figure 5.**
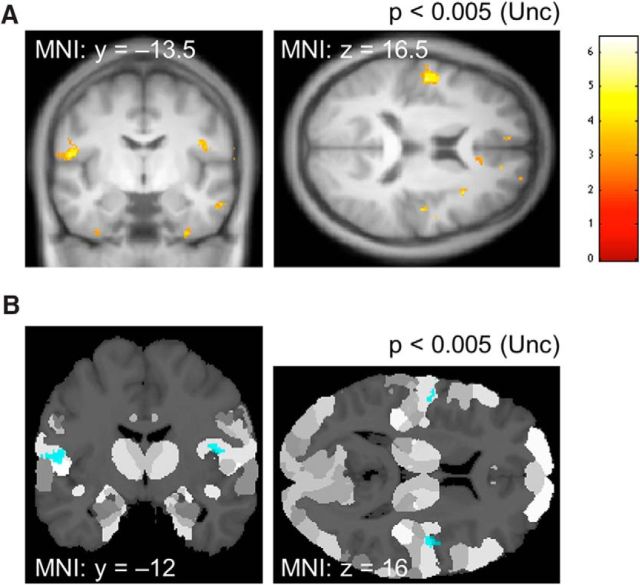
VBM changes in cold-trained versus warm-trained subjects. ***A***, Coronal and axial sections at an uncorrected threshold of *p* < 0.005. For the left cluster, peak MNI coordinate, *t*-statistics *p*-value, and spatial extent were [−56, −6, 13], respectively, *t* = 5.19, *p* = 0.00002, and 161 voxels; FWE correction within SII and PI was significant at *p* = 0.032; extent, 7 voxels. For the right cluster, corresponding statistics were [45, −13, 18], respectively, *t* = 6.43, *p* = 0.000001, 68 voxels, with FWE correction *p* = 0.0031; extent, 15 voxels. ***B***, The maximum probability map (MPM) (Collins et al., 1994) at the same threshold as ***A*** illustrating bilateral SII within anatomically defined masks of the two ROIs: SII (Eickhoff et al., 2005) and PI (Kurth et al., 2010). Localization probability (Eickhoff et al., 2005) was as follows: left cluster, 52.8% in area OP4, 14.1% in area TE 1.2, 8.4% in area TE 3; right cluster, 42.7% in area OP3 and 10.9% in area OP4.

In the cold-trained subjects, we observed symmetrical increases in VBM GM signal in SII, illustrated in Figure 5 at an uncorrected threshold of *p* < 0.005. This survived correction for multiple comparisons using the bilateral SII and PI cortex ROI mask. Based on the anatomical atlas, this increase in GM volume fell primarily with areas OP4 and OP3 (see figure legend for details).

**Figure 6.**
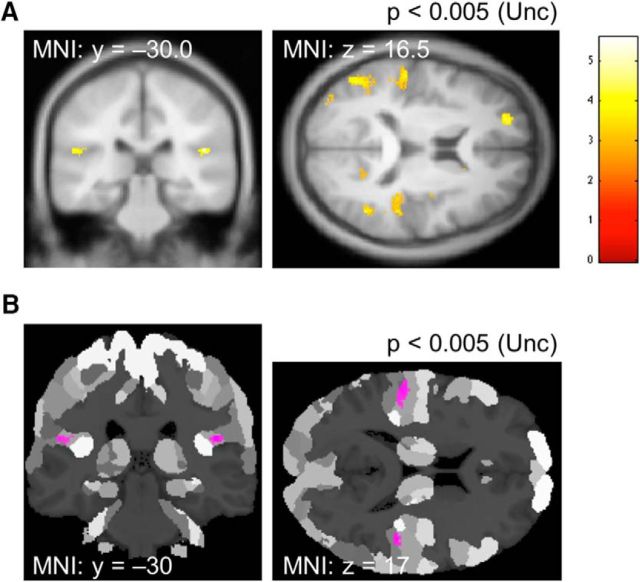
VBM changes in warm-trained versus cold-trained subjects. ***A***, Coronal and axial sections at an uncorrected threshold of *p* < 0.005. In the left cluster, peak MNI coordinate, *t*-statistics and *p-*value, and extent were [−57, −27, 15], respectively, *t* = 3.39, *p* = 0.00035, and 157 voxels, with nonsignificant FWE correction of *p* = 0.273. In the right cluster, there were two peaks, with corresponding stats: [48, −30, 17], respectively, *t* = 4.46, *p* = 0.0001, and 40 voxels; and at [38, −7, 12], respectively, *t* = 3.13, *p* = 0.003, and 16 voxels. FWE corrections yielded *p* = 0.12 and *p* = 0.756 respectively. ***B***, Maximum probability map (MPM) shown at the same threshold as ***A***. On the left, probabilistic localization was 96.5% in left area OP1 (SII) and 0.7% in left area PFcm (IPL). On the right, the caudal and rostral clusters had corresponding localization probabilities of 97.6% in area OP1 (SII) and 1.6% in area PFcm (IPL) (caudal right); and 97.8% in right area OP3 [VS], respectively.

In the warm-trained subjects, we identified symmetrical increases in GM volume in more posterior region of parietal opercular cortex at an uncorrected threshold of *p* < 0.005 (Fig. 6). Probabilistic anatomical localization isolated these areas as primarily within area OP1 (see figure legend). Some caution should be noted, however, because this result did not quite reach significance when corrected for multiple comparisons across the SII and PI cortices.

It could be argued that SI might also be expected to show temperature-specific responses, so in a supplementary analysis, we applied an SI mask (Geyer et al., 1999) and repeated the analysis above. In a posttraining minus pretraining contrast across all subjects, a just-significant peak was identified in right SI cortex (1 voxel at coordinates: 44, −34, 45), but we found no significant differences in the temperature-specific contrasts. In addition, we considered whether there might be laterality differences in the VBM data in SII. The study is underpowered to look directly at anatomical effects of laterality within the trained temperatures primarily because of the asymmetry of the brain in this region. However, we did perform an ROI analysis of the effect sizes of contralateral versus ipsilateral within masks defined by the cold and warm regions presented above. However, this did not identify significant differences: cold proportional increase, contralateral = 0.00863 and ipsilateral = 0.00583 (*p* = 0.55); warm proportional increase, contralateral = 0.00250 and ipsilateral = 0.00110 (*p* = 0.66).

**Figure 7.**
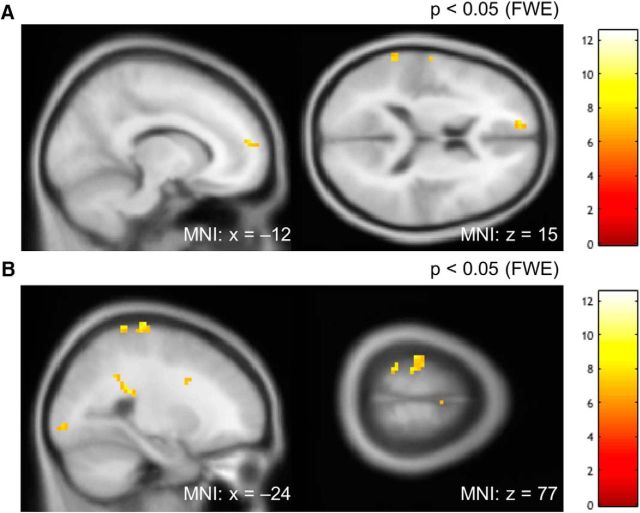
Seed-based correlation analysis of the rsfMRI. ***A***, Sagittal section at *x* = −12 and axial section at *z* = 15 at a whole-brain FWE-corrected threshold of *p* < 0.05. The peak coordinate, its *t*-statistics and *p-*value, and the extent of the cluster in the rostral medial prefrontal cortex were [−12, 57, 15], respectively, *t* = 7.96, *p* = 0.003, and 9 voxels. ***B***, Sagittal section at *x* = −24 and axial section at *z* = 77 at a FWE-corrected threshold of *p* < 0.05. The peak coordinate, its *t*-statistics and *p-*value, and the extent of the cluster in the post central gyrus (primary sensory cortex) were [−24, −27, 78], respectively, *t* = 9.57, *p* = 0.0001, and 28 voxels. We also noted responses in occipital lobe: [−27, −93, −3], respectively *t* = 8.03, *p* = 0.003, and 13 voxels.

We also acquired rsfMRI data before and after training to identify whether a broader network of regions might be involved in perceptual learning. This analysis is more exploratory because there are few prior studies on which to inform which brain regions might be involved in upstream/downstream aspects of fine temperature discrimination. With this in mind, we looked across all subjects using a seed defined by the VBM results (the anterior bilateral SII region for cold-trained subjects, and the posterior SII region for the warm-trained subjects). Specifically, we looked across all subjects to identify increases in connectivity in posttraining compared with pretraining scans and used a whole-brain FWE correction. This analysis identified increased connectivity in postcentral gryus, medial prefrontal cortex, and a region of visual cortex (looking purely at warm- or cold-trained groups alone did not identify brain regions surviving whole-brain FWE correction; Fig. 7).

## Discussion

The data provide three new findings about human discriminative thermosensation. First, we show that fine, subdegree discrimination of temperature can be enhanced through perceptual learning with repetitive training over a period of days. Second, we show that this improvement in performance is temperature specific (i.e., cool vs warm), indicating a functional dissociation within thermosensation. Finally, we show that perceptual learning correlates with putatively anatomically distinct temperature specific modules in SII cortex.

The debate about thermosensory cortical localization has tended to focus on data of neural responding to coarse-grained thermal stimuli at the cost of clearly defining the information-processing function of cortical regions. Discrimination is the prototypical function of primary sensory cortex across modalities. In vision, for instance, perceptual learning for orientation has been shown to involve primary visual cortex (Shibata et al., 2011). In thermosensation, although relatively computationally undemanding compared with other modalities, acuities of 0.3°C or less must almost certainly require both heterogeneity in the thermal response profiles of peripheral thermoreceptors and inference over a broad population of such thermoreceptors in the cortex.

The finding of dissociable modules for warm and cold discrimination in SII suggests that these pathways remain at least partially distinct, not only in peripheral nerve, spinal projection, and thalamus (Burton et al., 1979; Bushnell et al., 1993; Chen et al., 1996; Lenz and Dougherty, 1998; Yarmolinsky et al., 2016), but also in the cortex. Compatible with this functional dissociation, it has also been observed that putatively enhancing cold responses into the warm domain using menthol does not improve discrimination, suggesting that people cannot integrate warm and cold afferents spontaneously to improve discrimination (Barber et al., 2017). However, although warm and cold responses can be dissociated, this does not necessarily mean that they are independent and it remains entirely possible that warm-responsive afferents can contribute to cold discrimination and vice versa (Pogorzala et al., 2013). In particular, we did not include a test condition in which warm baseline temperatures were reduced or cold temperatures were increased (primarily because of the prohibitive duration of the test sessions). Therefore, we do not know, for instance, if training on temperature reductions from a cool baseline would generalize to increases from a cool baseline or decreases from a warm baseline.

Across both behavioral and imaging results, perceptual learning for cold temperatures appeared more robust. This may be unsurprising because the presumed dependence of warm discrimination primarily on unmyelinated C-fiber afferents compared with myelinated A-delta afferents for cold discrimination would suggest lesser fidelity of afferent information transmission (Craig et al., 2000; Bautista et al., 2007; Ran et al., 2016). There are other functional differences in these pathways: cold-responsive spinal cord neurons, which receive input from TRPM8-expressing dorsal-root ganglion (DRG) neurons, tend to show more adaptation to baseline temperature, which may allow them to respond more sensitively to small temperature changes in contrast to warm sensitive spinal neurons, which receive input from TRPV1-expressing DRG neurons. Thermosensing TRPM8 receptors may contribute to this adaptivity by showing baseline adaption response properties (Fujita et al., 2013). Peripheral pathways are also complicated by the fact that some afferents respond to both warming and cooling (Ran et al., 2016) and their contribution to discrimination is unclear.

Our study was not sufficiently powered to study the functional anatomy of the lateralization of thermosensory learning. Behaviorally, there was some suggestion, primarily in the cold domain, that learning was lateralized; that is, we did find a temperature × laterality interaction in the improvement of accuracy (*d*′). However we were not able to demonstrate this with an ROI approach to the imaging data. It remains a reasonable prediction that laterality-specific changes might be found in a larger sample size, although it should be noted that there is evidence that thermal responses may involve bilateral representations to a certain extent (Robinson and Burton, 1980).

Our results require rationalization with the clear evidence of graded thermal responses previously observed in insula cortex. One possibility is that insula acts in a behaviorally sensitive manner and reflects the homeostatic value of thermal input. That is, that insula integrates motivationally important information with sensory information to generate motivational values that can be used to guide behavior such as approach and avoidance. This would predict, for example, that insula representations of thermal stimuli would be dependent on current homeostatic state and that, for example, a cooling stimulus would have a different representation depending on whether an individual was hot (when it is rewarding) than cold (when it is aversive) (Hendersen and Graham, 1979). If confirmed, this would imply a functional dissociation between discriminative and homeostatic cortical loci in SII and insula, respectively.

The use of VBM allows a relatively unambiguous method to localize function under the assumption that evidence of modality-specific behavioral plasticity would be predicted to have a corresponding change in GM plasticity. Experience-dependent GM changes are unlikely to reflect fundamental changes in neuronal populations, but rather subtle changes in neuronal morphology, glial cell structure, vascularization, and signaling pathways (Zatorre et al., 2012). In the context of perceptual learning, it has several advantages over other neuroimaging methods and so provides a valuable complement to existing results. For example, BOLD fMRI responses can be confounded by large vessels and changes in the hemodynamic response function. Furthermore, simply observing BOLD responses opens awkward possible confounds, in particular interference from the explicit memory and thus attention arising from recall of training. In contrast, VBM effectively integrates over the history of perceptual learning in the absence of requirement to perform a task during evaluation of the brain. Furthermore, the use of an unreinforced paradigm (no feedback is given to the subjects about their performance) removes other confounds such as reward conditioning.

The resting-state network analysis identifies regions that might have a functional role in supporting perceptual learning. Although the nature of that function is speculative, two regions are noteworthy. First, postcentral gyrus activity might suggest connectivity with thermal representations in SI, although the region is not clearly within the usual topographic region of the leg. Therefore, the question of whether the thermosensitive input to SII comes directly from thalamus or indirectly from SI (both pathways exist anatomically) cannot be answered in the current study. The activity in medial PFC has been implicated in metacognitive evaluation of perceptual discrimination and might support a similar role here. Interestingly, metacognitive judgments can be dissociated from discriminative performance in thermal discrimination by application of menthol (which reduces accuracy but increases confidence in intermediate temperatures; Barber et al., 2017), so this hypothesis may be testable in the future.

Finally, our findings inform a parallel debate about the localization of nociceptive cortex with a similar and longstanding discussion about the relative importance of somatosensory and insula cortices. There is sufficient evidence that nociceptive sensation involves fine-discriminative processing to imply cortical processing (Mancini et al., 2012) and perceptual learning has been observed recently for nociceptive stimuli (Mancini et al., 2016). It is even possible that there might be different loci for different submodalities of pain (heat, cold, mechanical, inflammatory pain, etc.). However, the importance of nonpainful temperature processing is illustrated in the multiple interactions between pain and temperature, not least in chronic pain conditions such as poststroke pain, which is thought to arise through an imbalance between different spinothalamic pathways (Craig, 2003).
